# Backprojection Wiener deconvolution for computed tomographic reconstruction

**DOI:** 10.1371/journal.pone.0207907

**Published:** 2018-12-18

**Authors:** Zhenglin Wang, Jinhai Cai, William Guo, Martin Donnelley, David Parsons, Ivan Lee

**Affiliations:** 1 Centre for Intelligent Systems, School of Engineering and Technology, Central Queensland University, North Rockhampton, QLD, Australia; 2 School of Information Technology and Mathematical Sciences, The University of South Australia, Mawson Lakes, SA, Australia; 3 Respiratory and Sleep Medicine, Women’s and Children’s Hospital, North Adelaide, SA, Australia; 4 Robinson Research Institute and Adelaide Medical School, University of Adelaide, North Adelaide, SA, Australia; North Shore Long Island Jewish Health System, UNITED STATES

## Abstract

Analytical CT reconstruction is popular in practice because of its computational efficiency, but it suffers from low reconstruction quality when an insufficient number of projections are used. To address this issue, this paper presents a new analytical method of backprojection Wiener deconvolution (BPWD). BPWD executes backprojection first, and then applies a Wiener deconvolution to the whole backprojected image. The Wiener filter is derived from a ramp filter, enabling the proposed approach to perform reconstruction and denoising simultaneously. The use of a filter after backprojection does not differentiate between real sampled projections and interpolated ones, introducing reconstruction errors. Therefore a weighted ramp filter was applied to increase the contribution of real sampled projections in the reconstruction, thus improving reconstruction quality. Experiments on synthetic data and real phase-contrast x-ray images showed that the proposed approach yields better reconstruction quality compared to the classical filtered backprojection (FBP) method, with comparable reconstruction speed.

## Introduction

Reconstruction algorithms play an important role in Computed Tomographic (CT) imaging. Good reconstruction methods are required to yield high-quality CT slices, subsequently assisting practitioners and researchers to make better judgements, such as improved medical diagnoses. Reconstruction methods that are capable of generating high quality images with a small number of projections enable effective radiation dose reduction, which has significant benefits for human screening examinations, animal model research and for radiation sensitive samples [1]. On the other hand, improvements in acquisition hardware have enabled the resolution of volumetric CT projection images to increase, allowing the fine-details of the internal structures of the target to be visualised. For instance, phase contrast X-ray imaging setups at synchrotron facilities are capable of capturing high resolution images with pixel gaps of 10 *μ*m [2], with more than 2000-by-2000 pixels. In addition to the reconstruction quality, reconstruction methods should also consider computation resources (e.g., memory, CPU etc.) and reconstruction time. For these reasons, a large amount of research focused on improving the reconstruction quality has been conducted over the last few decades.

Current CT reconstruction algorithms can be classified into two classes: analytical methods and iterative methods. The analytical methods are mostly based on the projection-slice theorem, which holds in the continuous domain. To obtain a complete discrete image signal, the Nyquist—Shannon sampling theorem applies. With sufficient projection samples, analytical methods can consistently reconstruct high-quality images. In addition, analytical methods are computationally efficient and easy to implement [3]. Due to these advantages, the filtered backprojection algorithm (FBP), a popular analytical method, is usually used as the benchmark for assessing new reconstruction methods. However, the analysis in [4] reveals that *πN*/2 noiseless projections are required for reconstructing a CT image without any loss of information, where *N* denotes the number of detectors. In such a situation, more than 3000 projections are needed to recover a 2000-by-2000 pixel synchrotron CT image. This excessive demand for projections is considered a drawback for utilizing analytical methods in radiation sensitive applications including biological tissue studies.

To address the drawback of the analytical methods, iterative methods have been extensively studied. The iterative methods model the CT data acquisition directly in the discrete form as

$$\begin{array}{c} \begin{array}{c} p=Af+\epsilon , \end{array} \end{array}$$ (1)

where *p* denotes the acquired noisy projections, *A* denotes the CT imaging model named the system matrix, *f* is the target CT image, and *ϵ* represents the noise. The iterative methods have two important advantages. Firstly, *p* is a discrete representation which is the actual acquired data, so Eq (1) is more accurate to represent the real CT imaging model than the projection-slice theorem. Secondly, it is easy to incorporate prior knowledge into the inverse process of Eq (1) to improve the quality. The system of Eq (1) is likely under-determined or *A* is singular, so direct inversion, such as the least squares method, cannot be applied. In the early 1970s, Gordon *et al*. first proposed an algebraic reconstruction technique (ART) method [5] to solve Eq (1), which decomposed Eq (1) into a series of linear equations and calculated iterative solutions by projecting each projection onto a hyperplane. The previous solution was used as an initial “guess” for the successive projection. ART had a relatively rapid convergence speed but suffered from heavy salt and pepper noise [3]. The Simultaneous Iterative Reconstruction Technique (SIRT) [6] was then proposed to reduce the noise by introducing a set of correction terms. By combining ART and SIRT, the simultaneous algebraic reconstruction technique (SART) [7] applied a longitudinal Hamming window to emphasize the corrections applied near the middle of a projection ray, relative to those applied near its end, to further improve the reconstruction quality. On the other hand, SIRT and SART required more iterations to converge than ART. While the above three methods disregarded the noise term *ϵ*, recent approaches usually applied a regularization term (or prior term) to reduce the noise effect [4]. Examples include [8–12]. These methods can yield noticeable image quality improvements over FBP, at the cost of more iterations to converge to an optimal solution. Although numerous efforts have been made to develop various iterative methods with low computational complexity [13–15], they all suffered from significantly higher computational complexity than FBP. According to statistical analysis in [16], iterative methods usually have about two to three orders of magnitude larger computational cost than that of FBP. Iterative methods also require high computational resources, i.e., the system matrix *A* contains more than 10^12^ float numbers for solving a 2000-by-2000 pixel image, and are not robust across different protocols and applications, because their reconstruction performance is sensitive to the particular choice of parameters, which are hard to fine-tune [16–18]. These iterative reconstruction challenges have attracted substantial interest, with the use of GPU technology to speed up the computation [13, 19], cutting-edge optimization methods to increase convergence speed [14, 20], and the development of a rotation-based projector to avoid the occurrence of a large system matrix [21]. However, with the parallel development of higher-resolution detectors for performing volumetric CT scans these issues will likely remain.

To avoid the above issues, analytical methods are revisited in this paper. In contrast to the popular FBP, another analytical method is backprojection-then-filtering (BPF), first proposed by Bates *et al*. [22] in 1973. BPF works in the reverse order to FBP, reconstructing the images via first backprojecting, and then filtering the backprojection with a 2-dimensional (2D) ramp filter in the frequency domain. In theory, both methods achieve the same result [23] for continuous signals. In practice, projections are angularly sampled and discrete. The 2D ramp filter for BPF has to be formed via sampling a continuous ramp filter, resulting in a direct current (DC) shift and aliasing artifacts [18, 24, 25]. In addition, the projections are distributed in the polar coordinate system while the reconstructed image is in the Cartesian system, so an interpolation must be employed at the backprojection step to convert between coordinate systems. Usually, the nearest-neighbor interpolation is adopted, which inevitably leads to errors. If the common 2D ramp filter is applied, the interpolation error will be magnified after filtering. This impact could be even worse if the number of projections is insufficient. Although FBP suffers from similar noise, its filtration is performed prior to backprojection such that it can avoid magnifying the noise. Due to these factors, BPF does not perform as well as FBP in practice.

To overcome the shortcomings of BPF, a backprojection Wiener deconvolution (BPWD) approach is presented here. BPWD executes the backprojecting first, followed by a Wiener deconvolution instead of simple filtering. The point spread function for BPWD is derived from a 2D ramp filter, enabling it to reconstruct the target image. Meanwhile, the Wiener filtering inherent in deconvolution reduces the noise effect and improves the image quality. Furthermore, a weighted ramp filter, motivated by the projection geometry, is proposed for sparse-view CT applications. All the three methods have a common backprojection operation. Backprojection is considered the computational bottleneck of FBP or BPF [26], so the computational cost of BPWD is likely to be comparable to FBP or BPF. Table 1 compares the difference of the three analytical methods. These three methods are described and compared in terms of both reconstruction quality and computational cost, and their application to large synchrotron-based CT reconstructions is described. However, the proposed approach can be used for medium-size CT images as well.

**Table 1:** Difference of three backprojection based methods.

Step	FBP	BPF	BPWD
1	1D filtering for each projection	Backprojection	Backprojection
2	Backprojection	2D filtering for entire image	Wiener deconvolution for entire image

## Materials and methods

**Fig 1:**
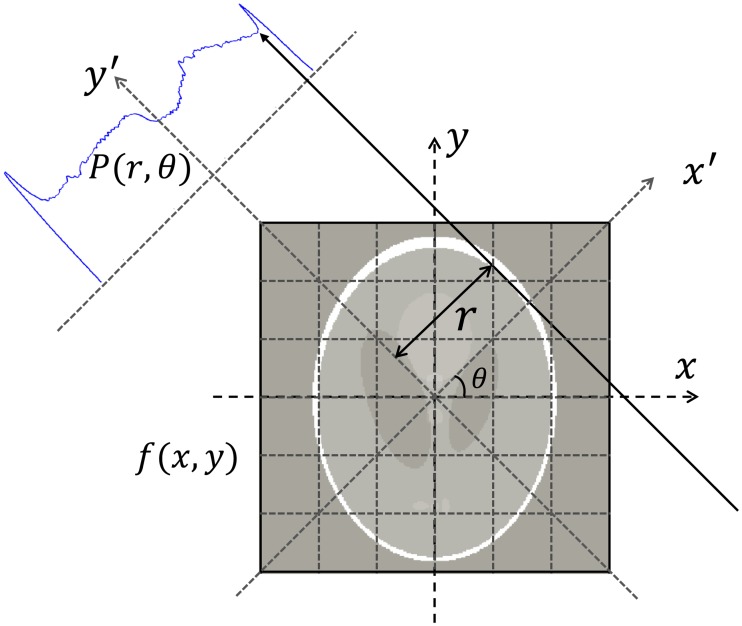
Parallel beam geometry. A one-dimensional detector-bin measures an integral of attenuation along the line at angle *θ* and distance *r* to the isocenter.

Long synchrotron beamlines designed for phase-contrast X-ray imaging (PCXI) and CT, such as the Imaging and Medical Beamline (IMBL) at the Australian Synchrotron, all utilise a parallel geometry. In a parallel geometry, each projection of an object at a given angle is seen as a set of line integrals (see Fig 1). In the Cartesian system (*x*, *y*-axes), a line at angle *θ* and distance to the isocenter *r* can be expressed as

$$\begin{array}{c} \begin{array}{c} x\cos\theta +y\sin\theta -r=0. \end{array} \end{array}$$ (2)

Let *f*(*x*, *y*) denote the pixel value at point (*x*, *y*), which usually represents the attenuation coefficient in X-ray CT applications. Then, the *θ*-view projection along the line of Eq (2) can be expressed as

$$\begin{array}{c} \begin{array}{c} p\left(r,\theta \right)=\int_{-\infty }^{+\infty }\int_{-\infty }^{+\infty }f(x,y)\delta (x\cos\theta +y\sin\theta -r)dxdy, \end{array} \end{array}$$ (3)

where *δ* denotes the 2D Dirac delta function. Eq (3) is also known as the Radon transform and the projection data is referred to as a sinogram. The backprojection of *p*(*r*, *θ*) is obtained by

$$\begin{array}{c} \begin{array}{c} b\left(x,y\right)=\int_{0}^{\pi }p(r,\theta )d\theta . \end{array} \end{array}$$ (4)

Simple backprojection of the projection images reconstructs a severely blurred image with a high density in the center, which is due to the fact that projections intersect at this area. To eliminate these effects, a filter is usually employed during reconstruction. In principle, the filtration can be applied before or after backprojection (FBP or BPF), and both methods can reconstruct the exact images if projections are noiseless and a sufficient number are acquired. However, as explained previously, in practice FBP is always superior to BPF due to the insufficient number of projections and the presence of noise. In this section, both FBP and BPF are briefly introduced and then BPWD is depicted.

### Filtered backprojection

FBP is a widely used technique to correct the blurring encountered in simple backprojection. It can be modelled by the following formula [27],

$$\begin{array}{c} f\left(x,y\right)=\int_{0}^{\pi }\int_{-\infty }^{+\infty }P\left(\omega ,\theta \right)\left|\omega \right|e^{i2\pi \omega (x\cos\theta +y\sin\theta )}d\omega d\theta , \end{array}$$ (5)

where *P*(*ω*, *θ*) is the 1D Fourier representation of the projection at *θ* and *ω* denotes the frequency. The inner integral can be regarded as a 1D inverse Fourier transform of the product *P*(*ω*, *θ*)|*ω*|, which represents a projection filtered by a 1D filter whose frequency representation is |*ω*|. The filter is commonly known as the ramp filter. The outer integral performs backprojection. So, FBP consists of two steps: filtration and then backprojection.

The ramp filter attenuates low frequencies and passes high frequencies, such that high-frequency features are emphasized while the magnitude of low-frequency features is reduced. Then, a subsequent backprojection is used to reconstruct a clear image. As the common ramp filter has the side effect of passing and magnifying additive noise from projection data, several modified filters (i.e., Shepp-Logan, cosine, Hamming and Hann) were developed for a range of applications, to reduce the high frequency cut-offs to certain degree, achieving a good trade-off between noise removal and feature preservation. However, it is difficult to construct a perfect filter for all scenarios without prior knowledge of the noise distribution. When the target image contains a high degree of complexity, common modified filters do little to improve the reconstruction quality. In contrast to previous pre-defined filters, a dynamic data-dependent filter was proposed to minimize the projection errors [28]. The data-dependent filter improved the reconstruction quality, but still suffered from expensive computational cost because it was derived from the ART method and changed in each iteration. Furthermore, the reconstruction artifacts from FBP are prominent when the number of projections is inadequate.

### Backprojection then filtering

BPF is mathematically modelled by [23],

$$\begin{array}{c} F(u,v)=B(u,v)\cdot |R|, \end{array}$$ (6)

where *F*(*u*, *v*), *B*(*u*, *v*) are the 2D Fourier representations of the target image and backprojection reconstruction respectively, *u* and *v* denotes the frequency, (⋅) means point-wise multiplication, and |*R*| is a 2D ramp filter in the frequency domain, defined as

$$\begin{array}{c} \left|R\right|=\{\begin{array}{cc} \sqrt{u^{2}+v^{2}}, & (u,v)\neq (0,0);\\ 0, & (u,v)=(0,0). \end{array} \end{array}$$ (7)

BPF backprojects the projections first to produce a blurry reconstruction with a high density at the center, and then deblurs it via a 2D ramp filter.

In comparison with FBP, BPF is less popular because of its inferior reconstruction performance, although both of them have similar computational complexity. There are two root causes. First, the 2D ramp filter |*R*| suffers from DC shift and aliasing artifacts. The 1D ramp filter |*ω*| for FBP has similar issues, but they can be eliminated by executing the filtering in the spatial domain [25]. Second, the filter for BPF is in the Cartesian coordinate system while the projections sampled radially are in the polar coordinate system. This conflict introduces interpolation errors, and the post filtering in BPF further amplifies such errors. Although a few strategies have been proposed to rectify these issues, the improved BPF still underperforms compared to FBP [23, 24, 29].

### Backprojection Wiener deconvolution

**Fig 2:**
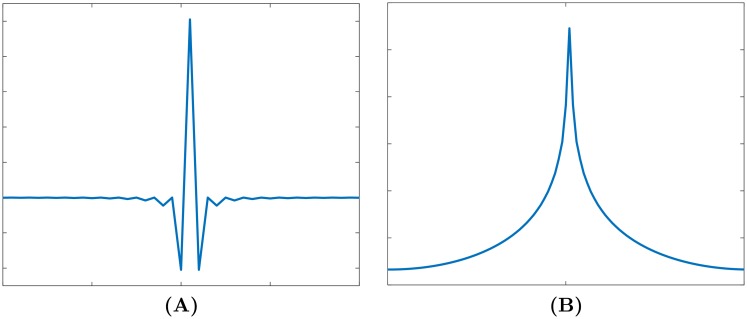
Ramp filter. **(A)** in the spatial space; **(B)** its corresponding PSF.

The convolution theorem states that a point-wise multiplication in the frequency domain corresponds to a convolution in the spatial domain. Thus, Eq (6) can be expressed as a convolution,

$$\begin{array}{c} f=b⊗r, \end{array}$$ (8)

where *r* is the spatial-domain ramp filter (see Fig 2a). Eq (8) can be used to solve *f* by convoluting *b* and *r* in the spatial domain. However, for finite-length discrete sequences of *b* and *r*, Eq (8) can obtain identical results to Eq (6) only when either *b* or *r* is periodic in line with the circular convolution theorem [30]. Eq (8) is more computationally expensive than Eq (6), so BPF is generally calculated via Eq (6).

In this subsection, a new method of BPWD is introduced. The definition of (7) shows that all the components in the 2D ramp filter |*R*| are nonzero except the center point which also causes the DC-shift issue. If a strategy is employed such that |*R*| contains no zero entries, i.e., the center point is set to a tiny value of machine epsilon, Eq (6) can be rewritten as

$$\begin{array}{c} B\left(u,v\right)=F\left(u,v\right)\cdot \frac{1}{\left|R\right|}. \end{array}$$ (9)

Again, by following the convolution theorem, (9) can be converted into

$$\begin{array}{c} b=f⊗h, \end{array}$$ (10)

where *h* is the spatial-domain representation of $\frac{1}{\left|R\right|}$. If noise is considered, Eq (10) can be generalized as

$$\begin{array}{c} b=f⊗h+n, \end{array}$$ (11)

where *n* denotes the unknown additive noise.

Eq (11) is a typical mathematical model for many imaging systems including CT, in which *h* is commonly known as the point spread function (PSF). An example is shown in Fig 2b. Numerous algorithms have been developed to recover *f* from Eq (11). Examples include Wiener deconvolution, the Lucy-Richardson algorithm and regularization. Wiener deconvolution applies a Wiener filter inherent in the deconvolution to the observed noisy samples, enabling simultaneous reconstruction and denoising. It is often conducted in the frequency domain to avoid the complex convolution operation. The Wiener filter is a pseudo-inverse filter of *h* in the frequency domain, expressed as

$$\begin{array}{c} G=\frac{1}{H}[\frac{H^{*}H}{H^{*}H+Z}], \end{array}$$ (12)

where *H* is the Fourier representation of *h* and H* denotes its complex conjugate, *G* is the frequency-domain representation of the Wiener filter, and *Z* is the noise-to-signal ratio in power spectrum domain. In the following context *σ* represents the corresponding spatial-domain value of *Z*. Then, the Fourier representation of the estimation of *f* can be calculated as

$$\begin{array}{c} \hat{F}=B\cdot G. \end{array}$$ (13)

When there is no noise, the Wiener filter is just the inverse of *H* which is the ramp filter. In other words, in the absence of noise BPWD is equivalent to BPF.

In real applications noise is inevitable, so Wiener deconvolution with an appropriate estimate of *σ* can minimize the noise effect and yield improved reconstruction quality. Although different CT scanners suffer from varying degrees of noise, the noise-to-signal ratio can be evaluated from previous experience. Users of clinical CT scanners usually have limited control over the reconstruction process, but Radiologists might adjust the noise ratio manually to obtain a subjectively optimum reconstruction for different clinical applications, if the reconstruction process was simple and fast. The same is true in a research environment for scientists using synchrotron beamlines, where there is a higher level of control over the choice of reconstruction parameters. In addition, Wiener deconvolution is a linear method to obtain a least squares solution, and no iterative process is needed. By contrast, the Lucy-Richardson and regularization methods have to involve an iterative process to converge to the optimal solution and require more reconstruction time. For this reason Wiener deconvolution performs the fastest and was adopted in the proposed framework.

### Weighted ramp filter

In FBP, filtering is an important step that improves the image quality. Different filters have different performance in terms of spatial resolution and noise removal. A sharp filter preserves spatial resolution but leaves noise, whereas a smooth filter yields polished images but loses fine edge details. The selection of the reconstruction kernel is therefore based on the specific clinical or research application. The ramp filter, deduced from mathematical analysis, is a typical sharp filter and able to maintain more high-frequency features. However, it has weak noise reduction performance, and even magnifies extraneous noise from projections. Variations of the ramp filter include Shepp-Logan, cosine, Hamming and Hann filters. Compared to the ramp filter, these derived filters lower the high-frequency cut-offs by different degrees for filtering out noise, but at the same time they introduce undesirable effects. The reconstructions of the ramp filter preserves sharp edges better, but presents more grainy noise than the other filters. The Hann filter has the lowest cut-off frequency, resulting in the lowest noise in the reconstruction, at the expense of texture blurring. The Shepp-Logan filter is a good compromise, and was therefore chosen for FBP in the following discussions. In BPWD, denoising relies on Wiener filtering, so the ramp filter was selected to derive the point spread function.

The projection slice theorem lays a foundation for analytical methods. It declares that when the projections are examined in the frequency domain, the corresponding Fourier samples locate only at the radial acquisition tracks, and the samples lying on other angles are vacant. This means that the samples reside in a polar coordinate system while the reconstructed image is represented in a Cartesian coordinate system. The conversion between different coordinate systems also causes misalignment. Both the vacancy and misalignment issues are usually solved at backprojection using a nearest-neighbor interpolation policy. However, interpolation errors are introduced and an insufficient number of projections worsens this type of error. In FBP, the filter is applied to each acquired projection directly, preventing noise from spreading to the interpolated samples. In BPF and BPWD, backprojection is executed before the filtration, and the interpolated samples accumulate both interpolation errors and acquisition noise. A common 2D ramp filter does not differentiate between the real acquired samples and the interpolated ones, resulting in a degraded reconstruction.

Intuitively, since the sampled projections contain less interpolation errors, they will contribute better accuracy in the reconstruction and should be given a higher weighting. Motivated by this idea, a weighted ramp filter was proposed in [31]. The weight matrix in their weighted ramp filter follows a *ρ*-norm shape (*ρ* = 0.6) to emphasize the contribution from the low-frequency samples because they are sampled relatively densely and introduce less interpolation errors. Their weighted ramp filter performs well for sparse-view CT reconstruction, but significantly underperforms when a sufficient number of projections are used.

In this section, a new weight matrix is proposed by following the projection trajectory. As a result, the proposed method can treat the real samples and the interpolated ones differently and increase the contribution of the former ones in the reconstruction. The weights are determined as below. It is assumed that there are *N* projections. The DC component in the frequency domain is sampled *N* times, so a weight of *N* is assigned to the central point. Similarly, if a frequency point is sampled only by one projection, its corresponding weight is 1; and if a frequency point is obtained through interpolation, its corresponding weight is set to 0. Thus, a weight matrix is constructed, denoted by *M*.

**Fig 3:**
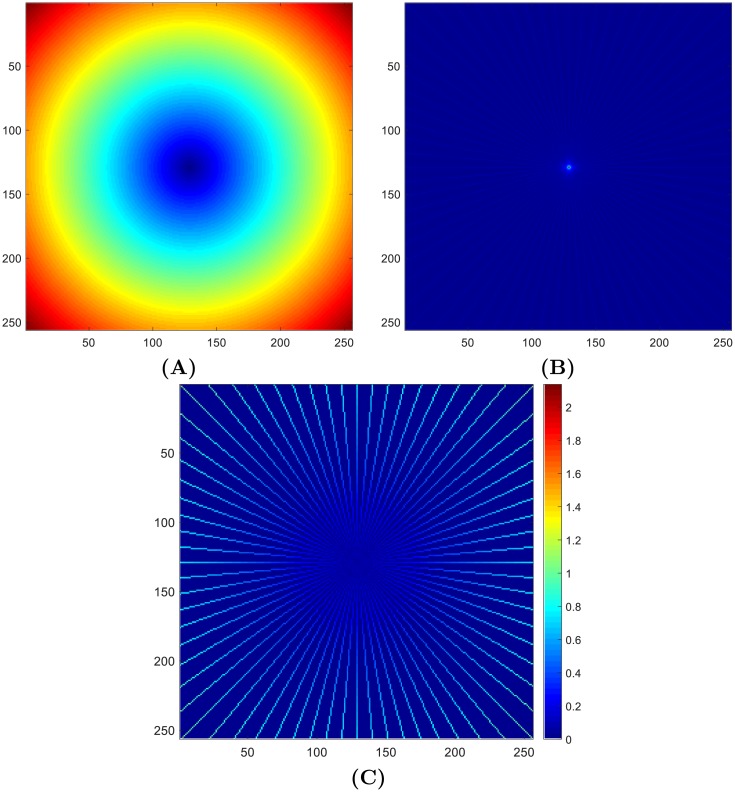
An example of constructing a weighted filter with 36 projections. **(A)** common ramp filter; **(B)** projection trajectories; **(C)** weighted ramp filter. To emphasize the visual effect of weight, the elements not on the projection trajectories are set to 0.

The proposed weighted ramp filter *W* is then expressed as

$$\begin{array}{c} W=(\alpha M+1)\cdot |R|, \end{array}$$ (14)

where *M* is a normalized weight matrix for real acquired projections and *α* controls their weights relative to interpolated samples which are supposed to have a base weight of 1. It is noted that *W* is identical to a common ramp filter when *α* = 0. An example is shown in Fig 3.

### Experiment setup

**Fig 4:**
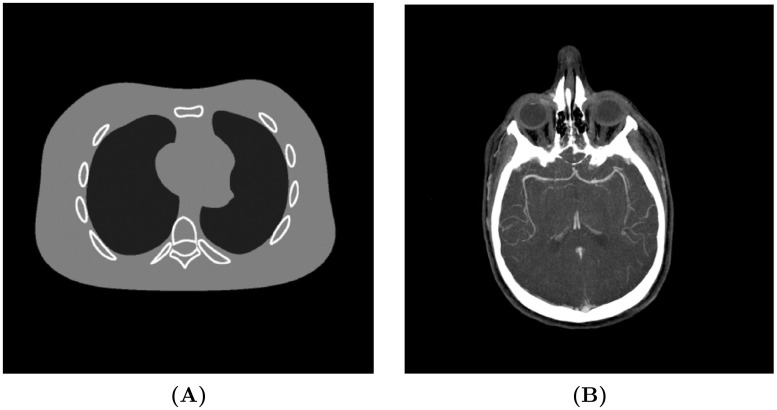
The two test images used in this paper. **(A)** NCAT phantom [34] with simple features; **(B)** real CT image with complex features. Both images are 2048 × 2048 pixels and normalized to a display window [0, 1].

Synchrotron propagation-based PCXI has been utilised for High-resolution visualization of airspace-containing organs in intact small animals by Parsons *et al*. [2], and in their research two potential requirements were raised: reducing the radiation dose for specialized diagnostic imaging studies at very high resolution in larger animals and potentially in humans, and flexibility of reconstruction methods allowing specified regions of interest (ROI) to be rendered with emphasis on different components of the tissue. The proposed method aims to allow the user to adjust the reconstruction parameters depending on their requirements for smoothness or sharpness of the textures in the ROI. Simulations were conducted to observe the performance of different parameter choices using the two test images in Fig 4. Synchrotron X-ray imaging data acquired at the Australian Synchrotron IMBL were then tested to provide a visual comparison and demonstrate the real-world performance of the proposed approach.

The data was collected under animal ethics approvals from the Women’s and Children’s Health Network (AE891-09-2013) and Australian Synchrotron (AS-2012-005) Animal Ethics Committees. The specimen was an adult mouse and the lungs were the primary organ studied. The animal preparation and imaging setup was as described in [32]. Briefly, mice were humanely killed by CO2 asphyxiation, and to minimise motion blur during scanning they were embedded in 2% agar while suspended in a 50 ml falcon tube with a 30 mm outside diameter. Monochromatic 30 keV X-rays and the Ruby beam monitor (consisting of a GadOx scintillator coupled to a pco.edge camera) were used with a sample-to-detector distance of 0.215 m. The field of view was set to 27.1 mm × 22.9 mm (2560 × 2160 pixels), and resulted in an effective pixel size of 10.6 × 10.6 *μ*m. An exposure length of 0.5 sec/projection was used, and a total of 1800 projections were acquired over 180 degrees (0.1 degrees/step).

BPWD was implemented in Matlab (version R2016a 64-bit, MathWorks) and tested on a laptop computer with an Intel Core-i7-6500U CPU and 16GB memory. A sparse-view test case was conducted by selecting 180 projections with equal intervals. Because there was no ground truth available, comparison was conducted based on visual appearance of the reconstructions. The synchrotron images were larger than 2000 × 2000 pixels. The ART and SART algorithms (i.e., open source implementations from https://github.com/phymhan/matlab-tomo-2d) produced a 2899 × 4194304 (90.6GB) system matrix and failed to run on our test system. Therefore, BPWD was only compared with analytical methods (FBP and BPF). The Shepp-Logan filter exhibits a good balance between noise reduction and preserving features, so it was adopted for FBP in simulation, yet in the real test applications reconstructions from FBP with ramp, Shepp-Logan, and Hann filters are presented to demonstrate the performance of high, middle and low cut-off frequency filtering. The common weighted 2D ramp filter was used for BPF and BPWD. BPF-W denotes BPF with the weighted ramp filter, and BPWD-W denotes BPWD with the weighted ramp filter. SNR is a popular measure to evaluate the quality of medical images [33] and was therefore used as the criterion to judge algorithm performance. The tunable parameters for the proposed weighted 2D ramp filter are *α* and *σ*, and their impact on reconstruction quality was investigated, along with a comprehensive comparison of the performance using both simulation and real CT data.

## Results and discussion

### Choice of *α* and *σ*

**Fig 5:**
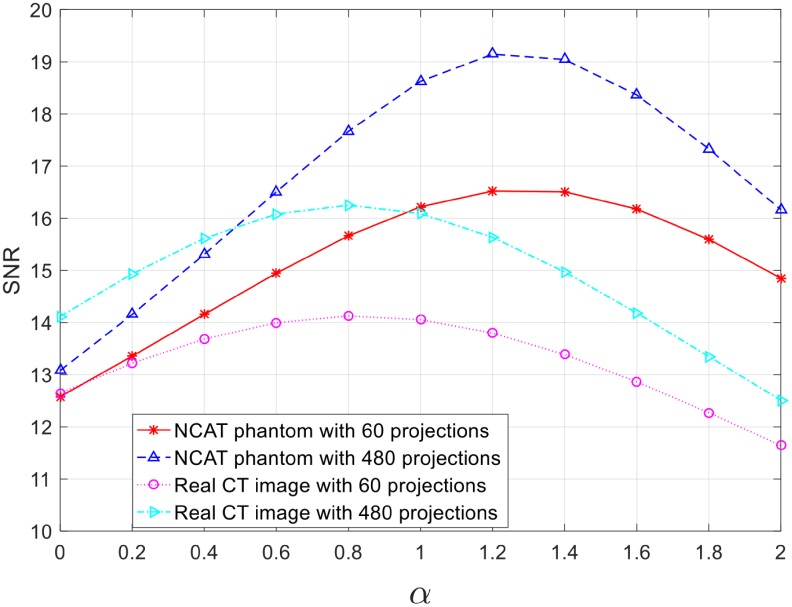
SNR performance with different *α*.

Fig 5 shows the performance trend with different *α*. Clearly, *α* affected the reconstruction quality in all test cases. For the NCAT phantom image with simple texture, the reconstruction achieved the best quality when *α* = 1.2 regardless of the number of projections. In comparison with a standard 2D ramp filter (*α* = 0), the proposed weighted 2D ramp filter obtained up to 3.94 dB gain in SNR for 60-projection reconstruction and 6.07 dB gain for 480-projection reconstruction. For a real CT image with complex texture, the best reconstruction quality was achieved at *α* = 0.8 in both 60-projection and 480-projection reconstructions, and the proposed weighted filter contributed an improvement up to 1.47 dB and 2.13 dB gain, respectively. The results reveal that (1) the choice of *α* is not susceptible to the number of projections; (2) the proposed filter is more favourable to CT reconstruction with simple texture and sufficient projections. In practice, it is difficult to obtain the optimal choice of *α* every time, so *α* was universally chosen as 1 for the following tests.

**Fig 6:**
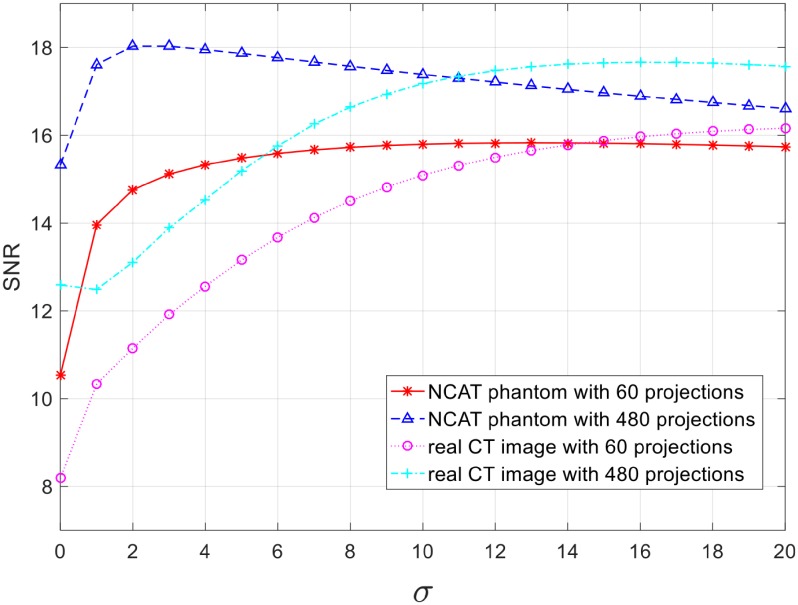
SNR performance with different *σ*.

The *σ* parameter estimates the noise level. In CT imaging, noise can come from scanning systems, detectors as well as the reconstruction procedure (i.e., interpolation errors). In this test, *σ* primarily results from the noise introduced by the use of an insufficient number of of projections, which is important to sparse-view CT imaging applications. The experimental results were assembled in Fig 6, and they showed that the choice of *σ* affects the reconstruction quality. In the test case of a real CT image with 60 projections, choice of an appropriate *σ* could improve the reconstruction quality with an SNR gain up to 8 dB in comparison to *σ* = 0 (equivalent to BPF). Larger *σ* was favourable when the number of projections was insufficient, but could degrade the reconstructed image quality when the number of projections was adequate. The phenomena reasonably reflect the fact that fewer projections cause more interpolation errors, which subsequently introduces higher levels of noise. Overall, the performance of *σ* was prone to both the image texture and the number of projections. It is difficult to seek a fixed *σ* that can achieve the optimal performance for all scenarios, but the experimental results revealed that an optimal *σ* would lie in the range of [1, 16]. A large *σ* reduces the noise but smooths out the fine details simultaneously, so a fixed value of *σ* = 7 was used in the simulation test while *σ* = 1, 7 and 15 tested in the real applications for varying purposes (i.e., examine bones or soft tissues) so as to allow radiologists or scientists to tune *σ* themselves to obtain the quality subject to their need.

### Numerical simulation

**Fig 7:**
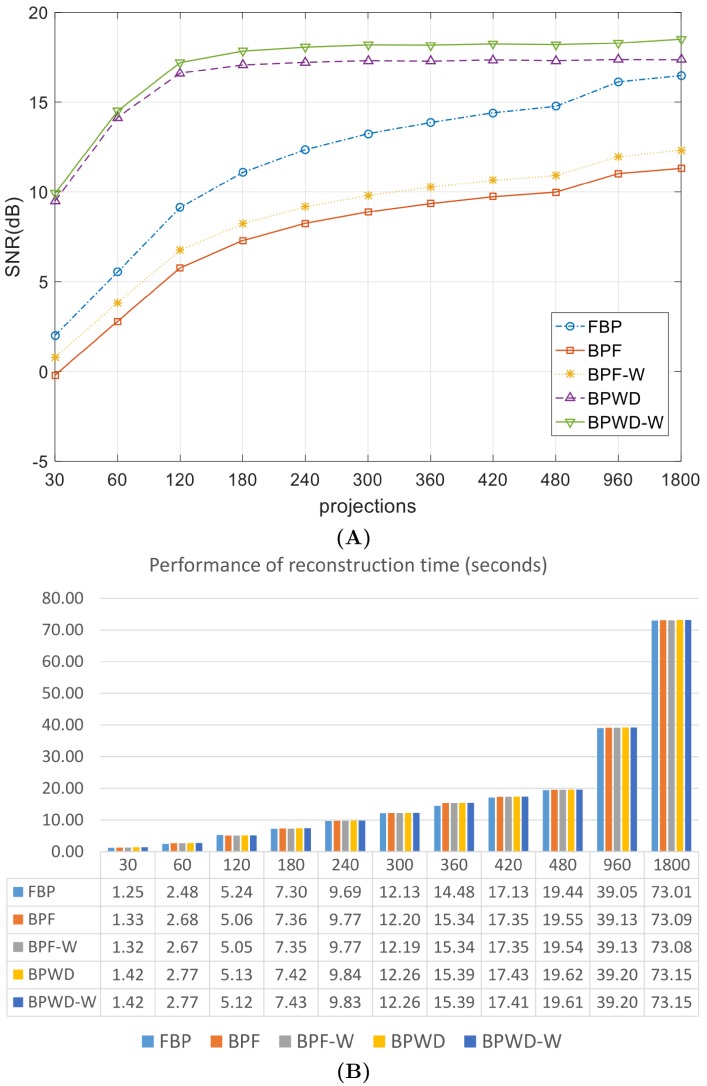
Performance comparison of different methods based on simulations. **(A)** SNR performance; **(B)** reconstruction time.

**Fig 8:**
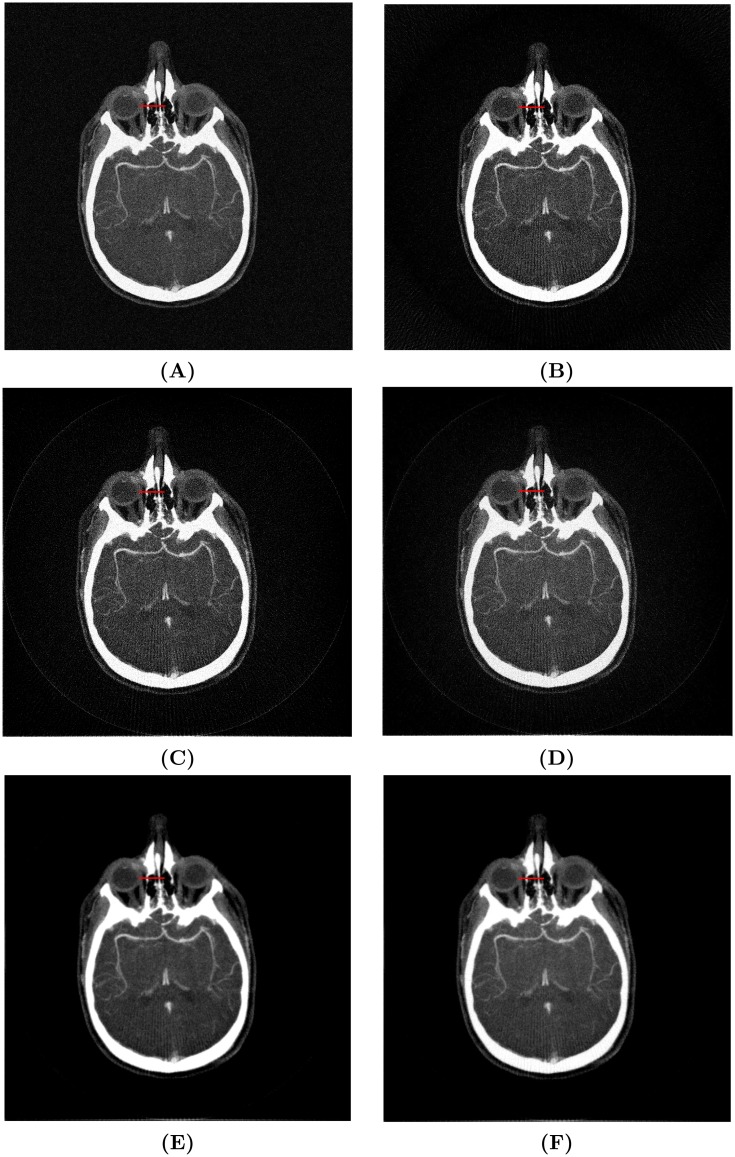
Visual comparison using 180 projections. **(A)** original image with Gaussian noise; **(B)** FBP; **(C)** BPF; **(D)** BPF-W; **(E)** BPWD; **(F)** BPWD-W.

**Fig 9:**
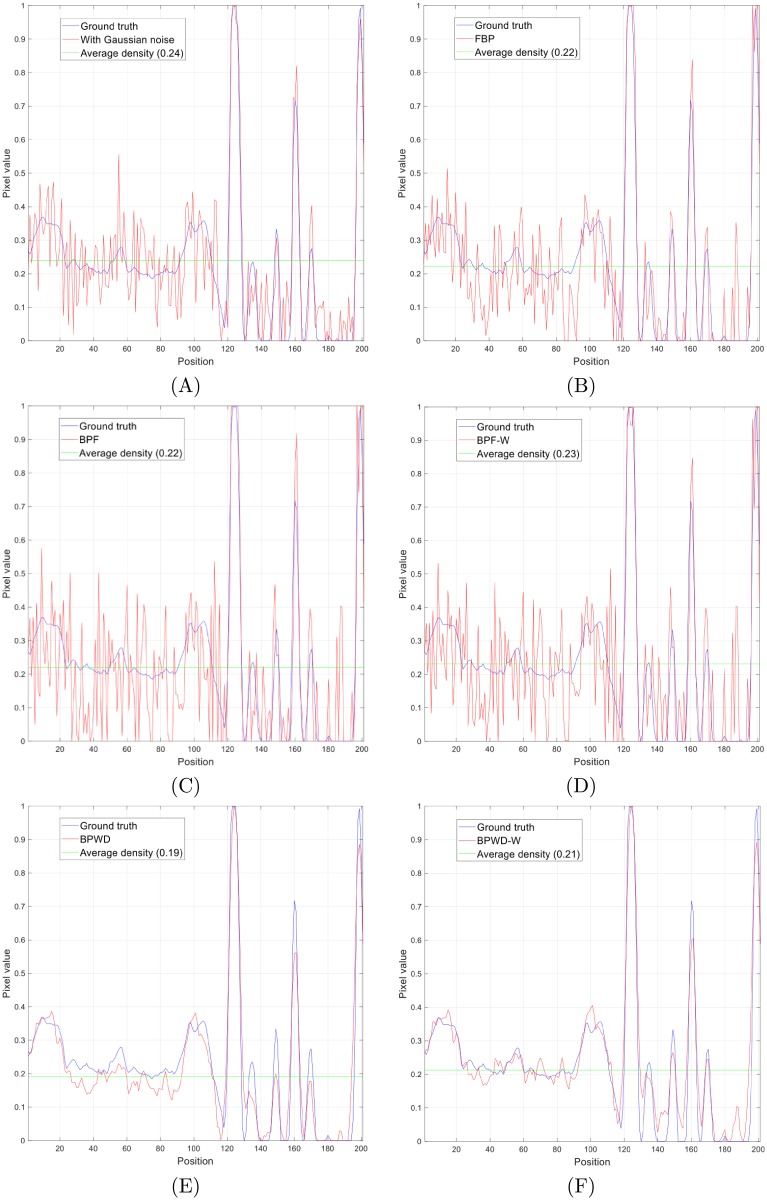
Partial line (indicated by red line in Fig 8) profiles (normalised to the same maximum value) using 180 projections. **(A)** original image with Gaussian noise; **(B)** FBP; **(C)** BPF; **(D)** BPF-W; **(E)** BPWD; **(F)** BPWD-W.

Numerical simulations were tested to provide both visual and quantitative comparisons. It is difficult to obtain noiseless projections in real CT applications, and the noise in common CT imaging systems was found to follow a Gaussian distribution [35, 36]. So, a Gaussian noise with zero mean and 0.01 variance was added to Fig 4b to approximate a real application. However, this prior knowledge was supposed to be unknown, so *α* and *σ* were set to the values suggested previously. The SNR performances were recorded in Table 2 and Fig 7a. Visual results were compared in Fig 8 and partial line profiles were compared in Fig 9.

**Table 2:** SNR (dB) performance of different methods.

Projections	FBP	BPF	BPF-W	BPWD	BPWD-W
30	1.91	-0.27	0.72	9.48	9.91
60	5.53	2.78	3.82	14.19	14.53
120	9.12	5.77	6.74	16.59	17.19
180	11.09	7.29	8.22	17.08	17.83
240	12.33	8.23	9.15	17.25	18.07
300	13.24	8.88	9.80	17.27	18.15
360	13.91	9.37	10.29	17.36	18.25
420	14.37	9.71	10.63	17.33	18.22
480	14.81	9.99	10.91	17.36	18.23
960	16.11	11.01	11.95	17.35	18.28
1800	16.44	11.30	12.32	17.33	18.48

The simulation results showed that the proposed approach outperformed BPF and FBP consistently. In comparison with BPF, BPWD performed a SNR gain from 9.69 dB for 30-projection reconstruction to 7.2 dB for 1800-projection reconstruction. In comparison with the popular FBP, BPWD also performed a SNR gain from 7.48 dB to 2.4 dB. Although the proposed weighted 2D ramp filter contributed a limited improvement with up to 1.1 dB, the gains were always positive. Interestingly, when the number of projections increased, the weighted filter could still improve the quality and help retrieve more high-frequency details. It was also observed that the proposed weighted filter could help BPF improve its reconstruction performance. Overall, BPWD with the proposed weighted filter (BPWD-W) always achieved the best performance in terms of quality, and BPF performed the worst. In addition, BPWD-W achieved a relatively high quality reconstruction of 17.5 dB with only 180 projections. After that, only 1.8 dB gain was achieved for an additional 1620 projections. By contrast, FBP achieved an SNR performance of 11.2 dB, and 1620 additional projections increased the SNR to 16.5 dB. Visual comparison was made in Fig 8. FBP removed the Gaussian noise mostly due to the Shepp-Logan filter. BPF performed poorly and even introduced interpolation errors. For example, in the reconstructions of Fig 8(c) and 8(d) a noisy circle was observed surrounding the target images. BPWD-W presented the best quality images. Its partial line profile showed a good fit with the ground truth. Although the SNR difference between BPWD and BPWD-W was minor, it was observed that the proposed weighted filter could preserve more image details. For example, a spike feature at Position 180 remained only in the BPWD-W reconstruction. It is also noted that all methods attenuated the mean density of the entire image, with FBP and BPF decreasing 8.3% and BPWD-W 12.5%. The loss in density could result in removal of fine details.

Since the computational cost of backprojection dominates in all the three methods of FBP, BPF and BPWD, their reconstruction times should be similar. To obtain fair comparisons, all the three methods were implemented using standard Matlab functions (i.e., *radon*, *iradon* and *deconvwnr*) and their reconstruction times were recorded in Fig 7b. The proposed weighted 2D ramp filter was pre-defined and pre-computed, so its construction time was not taken into account. The results were based on average values produced by 10 trials each. The experimental results verified that all the methods had nearly the same reconstruction speeds. BPWD took slightly more reconstruction time because the Wiener deconvolution was more complex than simple filtering. Unsurprisingly, the number of projections affected the reconstruction speed, from 1.4 seconds for a 30-projection reconstruction, to 73 seconds for a 1800-projection reconstruction. On the other hand, it is noted that all three analytical methods could deal with 2048 × 2048 pixel CT images with 1800 projections on an ordinary laptop, while iterative methods failed. The reconstruction time could be reduced if the algorithms were implemented with a different language such as C/C++ or if a GPU is utilized.

### Real CT scan data

Since FBP is unable to mitigate the noise a denoising process could be considered after reconstruction. Therefore, additional tests were used to investigate whether the 2D adaptive Wiener noise-removal filtering could further improve FBP reconstructions. However, such a strategy is in principle different from the proposed BPWD. Common Wiener filtering for denoising uses a pixel-wise adaptive Wiener method based on statistics estimated from a local neighborhood of each pixel. In other words, the Wiener filter varies over pixels within the image and needs to be calculated dynamically. So, this process will naturally be time-consuming if the window size is large. In addition to the need of estimating the noise level, the window size, which is vital to denoising performance, has to be guessed. An improper choice of window size will result in degradation of the image quality. By contrast, the Wiener filter in BPWD is derived from a ramp filter which is independent of specific images. Once an estimation of noise level is chosen, the Wiener filter is determined and then applied to the entire image through a deconvolution. So, the proposed BPWD method embeds Wiener filtering into the reconstruction process and is more computationally efficient. However, the reconstruction quality of BPWD-W, FBP and FBP with additional Wiener filtering were investigated and compared in the following sparse-view test. The noise level was unknown, so *σ* = 1, 7 and 15 were investigated for BPWD-W while the adaptive noise estimation policy was used for Wiener filtering of FBP. In addition, the window size for Wiener filtering of FBP used two guesses: 5 and 10.

**Fig 10:**
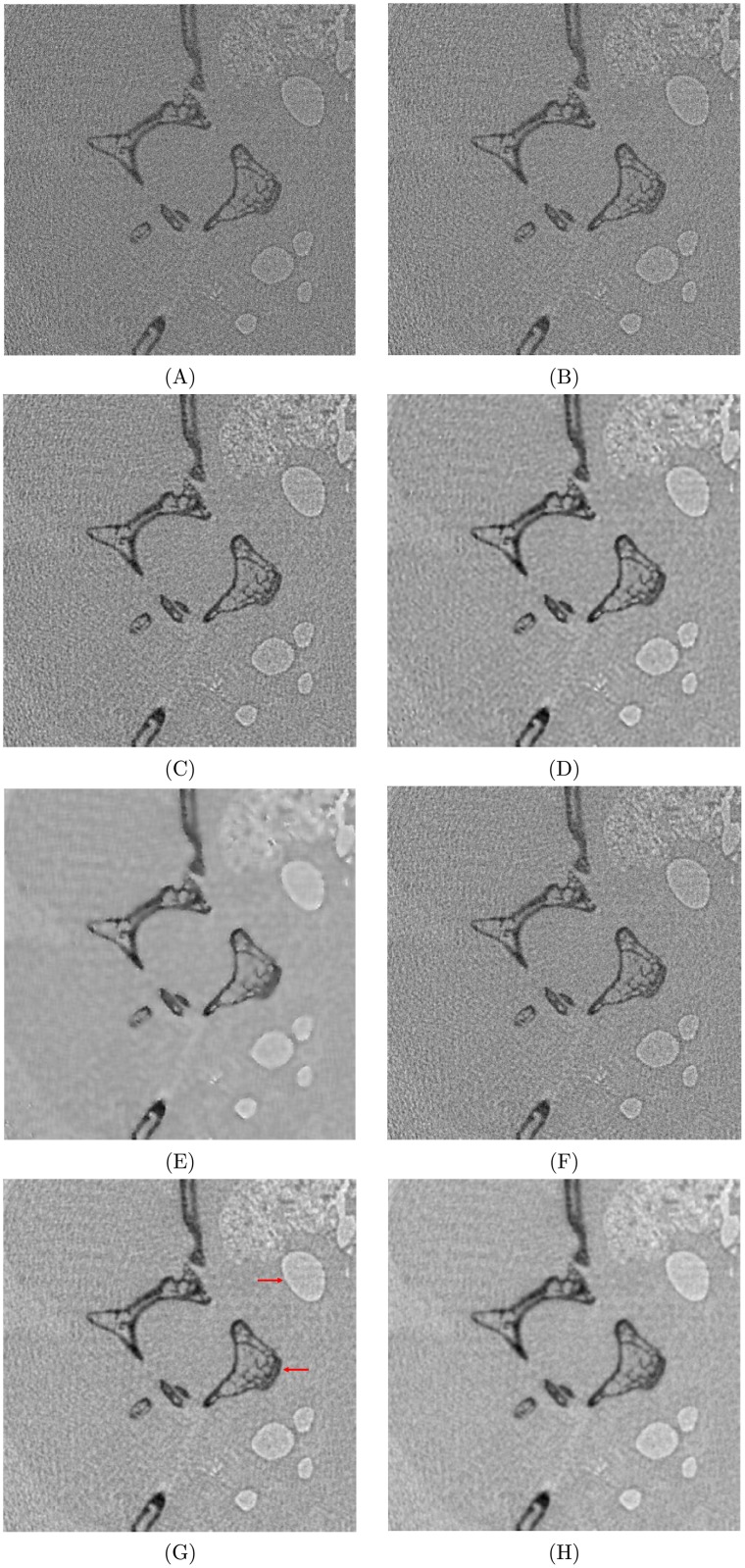
Sparse-view reconstructions with 180 projections from different methods. FBP with **(A)** ramp, **(B)** Shepp-Logan, or **(C)** Hann filter; FBP with Hann filter then Wiener noise-removal filtering with a window size of **(D)** 5 or **(E)** 10; BPWD-W with **(F)** *σ* = 1, **(G)** *σ* = 7, or **(H)** *σ* = 15.

Fig 10 recorded the reconstruction results from FBP, FBP-then-Wiener-filtering and BPWD-W, all performed with 180 projections. It was observed that BPWD-W with *σ* = 7 yielded reconstructions with less noise and better contrast than the other methods. In Fig 10G the airway and spine (both indicated by red arrows) were clearly contrasted from their background, while other reconstructions contained either severe grainy noise or blurry bone fine structure. Owing to its lowest cut-off frequency, the Hann filter with FBP achieved better contrast than the ramp and Shepp-Logan filters, but yielded a quality just comparable to BPWD-W with *σ* = 1. A post Wiener filtering could reduce the noise, see Fig 10(D) and 10(E) over Fig 10C, but introduced some noise-like sparkle spots. Moreover, an improper window size can blur the reconstruction (i.e., Fig 10E). Thus, FBP-then-Wiener-filtering might have the limitations: (1) the noise is estimated based on a local window, which might magnify a local spike noise as a valid signal; and (2) a proper window size is difficult to estimate.

**Fig 11:**
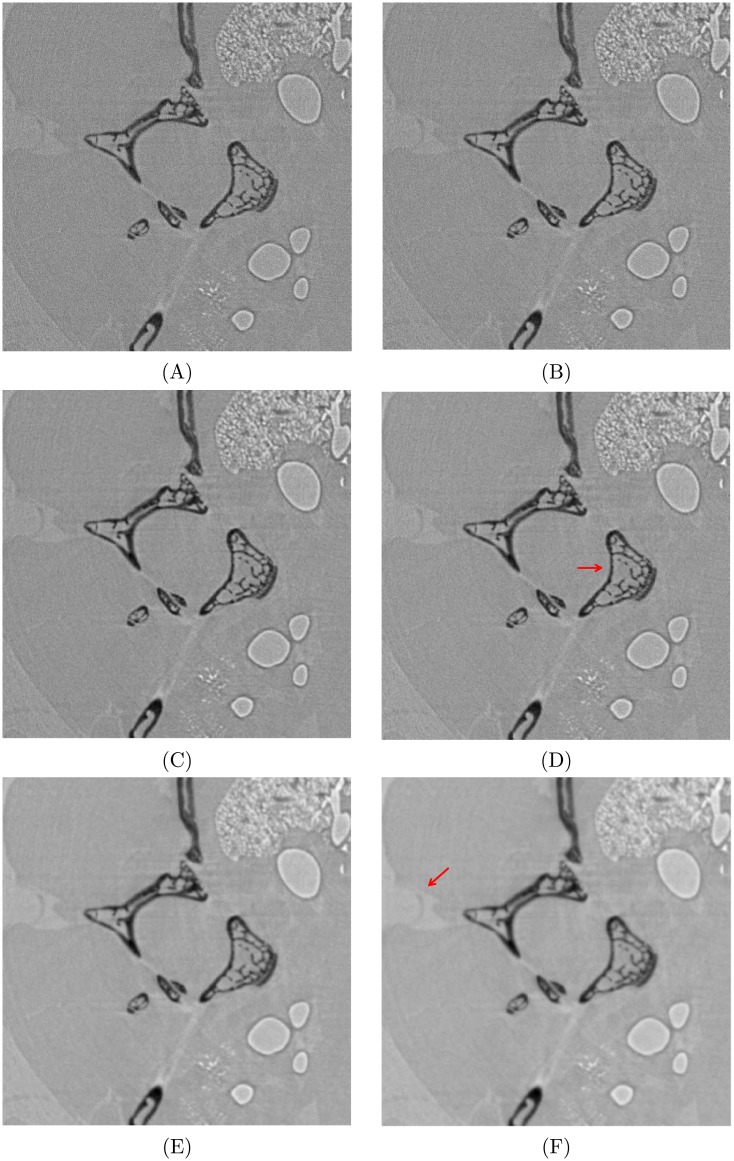
Comparison of reconstructions between FBP and BPWD-W with 1800 projections. FBP with **(A)** ramp, **(B)** Shepp-Logan, or **(C)** Hann filter; BPWD-W with **(D)** *σ* = 1, **(E)** *σ* = 7, or **(F)** *σ* = 15.

Scientists and researchers may expect to investigate more micro-scale details if there are sufficient projections provided, thus reconstructions with complete projections were examined and a small region is shown in Fig 11. With its denoising behavior, BPWD-W can offer more flexibility by choosing different *σ*. A smaller *σ* is helpful to produce a strong contrast for bone structure (Fig 11D), while a larger *σ* can provide a clearer image for soft issues (Fig 11F). By contrast, when the number of projections is large, reconstructions from FBP with different filters all have good performance in rendering the bone structures, but suffer from noise effect for soft tissues.

**Fig 12:**
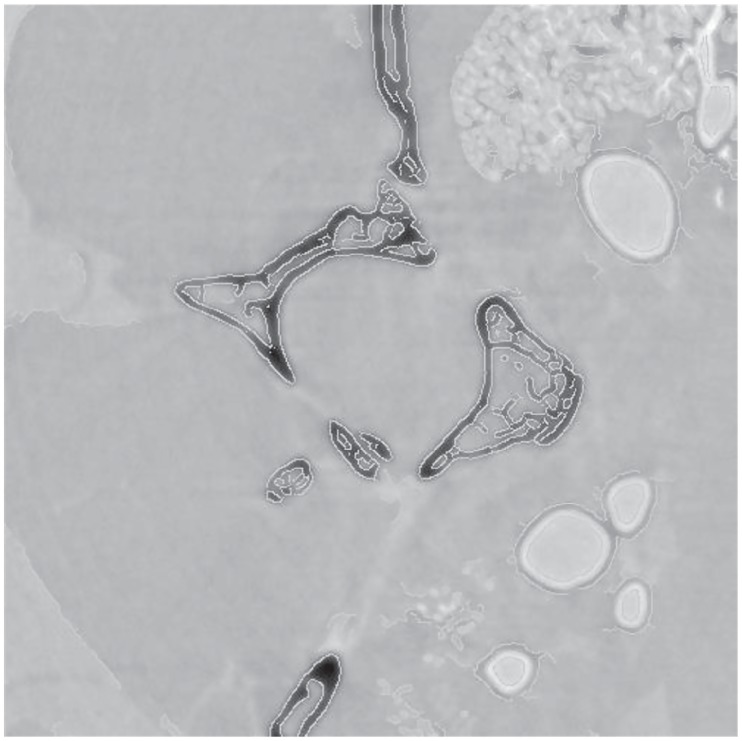
Canny edge enhancement using (Fig 11D and 11F). Note that the white fringes around the air containing structures are characteristic of the propagation-based synchrotron PCXI setup.

The above results showed that the values of *σ* affected the reconstruction quality. Once the time-consuming backprojection completes, the Wiener deconvolution can be accomplished within a few milliseconds. With the fast speed of Wiener deconvolution it is possible to make *σ* values dynamically adjustable by users. Furthermore, a post edge enhancement technique can be applied to combine the reconstructions from a small and big *σ* as

$$\begin{array}{c} \begin{array}{c} P=(1-cE)\cdot I+cE\cdot O \end{array} \end{array}$$ (15)

where *O* is the reconstruction with a small *σ* value, *I* the reconstruction with a large *σ* value, *E* the Canny edge information, and *c* a weight factor which is chosen as 0.1 empirically. Fig 12 shows an edge enhanced reconstruction, which presents a smooth texture but with strong edge information.

## Conclusion

In this paper, a new analytical method using backprojection and Wiener deconvolution was presented. The Wiener deconvolution embedded a denoising process within the reconstruction such that the proposed approach exhibited good performance in noise reduction. Inspired by the radial geometry of CT systems, a weighted ramp filter was proposed to further improve the reconstruction quality. Both the simulation and real data test illustrated that the proposed approach achieved substantially better performance in quality, and comparable performance in terms of reconstruction speed, than that of the classical FBP. However, whether or not the proposed BPWD can achieve a comparable reconstruction quality to the state-of-the-art iterative algorithms is important to explore in future studies.
